# Increased Microglia/Macrophage Gene Expression in a Subset of Adult and Pediatric Astrocytomas

**DOI:** 10.1371/journal.pone.0043339

**Published:** 2012-08-22

**Authors:** Jane R. Engler, Aaron E. Robinson, Ivan Smirnov, J. Graeme Hodgson, Mitchel S. Berger, Nalin Gupta, C. David James, Annette Molinaro, Joanna J. Phillips

**Affiliations:** 1 Department of Neurological Surgery, University of California San Francisco, San Francisco, California, United States of America; 2 Department of Pathology, University of California San Francisco, San Francisco, California, United States of America; 3 Department of Epidemiology and Biostatistics, University of California San Francisco, San Francisco, California, United States of America; 4 Brain Tumor Research Center, University of California San Francisco, San Francisco, California, United States of America; 5 Division of Oncology, Pfizer, La Jolla, California, United states of America; Institute of Cancer Research, United Kingdom

## Abstract

Glioblastoma (GBM) is a highly malignant brain tumor with a dismal prognosis. Gene expression profiling of GBM has revealed clinically relevant tumor subtypes, and this provides exciting opportunities to better understand disease pathogenesis. Results from an increasing number of studies demonstrate a role for the immune response in cancer progression, yet it is unclear how the immune response differs across tumor subtypes and how it affects outcome. Utilizing gene expression data from The Cancer Genome Atlas Project and the Gene Expression Omnibus database, we demonstrate an enrichment of immune response-related gene expression in the mesenchymal subtype of adult GBM (n = 173) and pediatric high-grade gliomas (n = 53). In an independent cohort of pediatric astrocytomas (n = 24) from UCSF, we stratified tumors into subtypes and confirmed these findings. Using novel immune cell-specific gene signatures we demonstrate selective enrichment of microglia/macrophage-related genes in adult and pediatric GBM tumors of the mesenchymal subtype. Furthermore, immunostaining of adult GBM tumors showed significantly higher cell numbers of microglia/macrophages in mesenchymal versus non-mesenchymal tumors (p = 0.04). Interestingly, adult GBM tumors with the shortest survival had significant enrichment of microglia/macrophage-related genes but this was not true for pediatric GBMs. Consistent with an association with poor outcome, immune response-related genes were highly represented in an adult poor prognosis gene signature, with the expression of genes related to macrophage recruitment and activation being most strongly associated with survival (p<0.05) using CoxBoost multivariate modeling. Using a microglia/macrophage high gene signature derived from quantification of tumor-infiltrating cells in adult GBM, we identified enrichment of genes characteristic of CD4 T cells, granulocytes, and microglia/macrophages (n = 573). These studies support a role for the immune response, particularly the microglia/macrophage response, in the biology of an important subset of GBM. Identification of this subset may be important for future therapeutic stratification.

## Introduction

Glioblastoma (GBM), WHO grade IV astrocytoma, is a highly malignant disease with a poor prognosis despite aggressive treatment strategies [1]. Results from molecular profiling studies of GBM suggest that stratification of these tumors into clinically relevant subtypes will lead to improved therapy outcomes as a result of individualizing treatment based on tumor molecular signatures. In adults, GBM is the most common primary malignant brain tumor. In children, high-grade astrocytomas, including GBM, are less common, comprising 8–12% of central nervous system (CNS) malignancies, but are equally heterogeneous and present with a similarly dismal outcome [2].

Gene expression profiling of adult GBM has greatly aided our understanding of underlying tumor pathogenesis [3]–[7], and has promoted GBM stratification into tumor subtypes [4], [7]. While many different subtype classifications have been performed, the mesenchymal subtype as defined by both Phillips et al. [4] and Verhaak et al. [7] shows the greatest concordance [8]. The mesenchymal GBM subtype is associated with reduced survival [4], and it is characterized by upregulation of genes involved in tumor microenvironment interactions and processes [4], [7], [9], [10].

Although adult and pediatric high-grade astrocytomas are widely considered as having distinct clinical and molecular characteristics, a recent gene expression profiling study identified three major subtypes of pediatric high-grade glioma that recapitulated subtypes identified by Phillips et al. in adult high grade astrocytoma [4]: HC1/proliferative, HC2/proneural and HC3/mesenchymal [11]. Similar to adult tumors, genes upregulated in the HC3/mesenchymal subtype were associated with extracellular matrix-receptor interactions and cell adhesion [11]. Given the subtype-specific increase in expression of genes involved in tumor microenvironment interactions, we reasoned that a subset of adult GBM and pediatric high-grade astrocytoma may have a pronounced and potentially important immune cell component.

Several lines of evidence suggest that the immune response is important to glioma biology. First, there is a significant inverse correlation between atopic disease and serologic markers of allergy, including IgE, and the sporadic incidence of glioma [12], [13]. Secondly, single nucleotide polymorphisms of immune system genes are significantly associated with glioma risk [14]. Lastly, manipulation of the immune response in experimental models for glioma alter disease outcome [15]–[18]. As a result of such observations, a number of novel immunomodulatory strategies are currently in clinical trials for treating GBM patients [19].

The predominant immune cell infiltrate in human GBM consists of CD45+CD11b+ microglia/macrophages [20], [21] and in experimental models of glioma, tumor-associated microglia/macrophages directly influence tumor growth [17], [18] and invasion [16]. GBM also elicits an adaptive immune response and there is an increased infiltrate of CD4+FoxP3+ regulatory T cells in GBM relative to lower-grade gliomas and normal brain [20], [22], [23]. These regulatory T cells are thought to contribute to the profound lack of activated effector CD8+ T cells in GBM [20] and their depletion in model systems of glioma have been shown to promote host survival [15].

Given the clinical and experimental evidence supporting a role for inflammation in GBM, we combined transcriptional expression profiling and immunoprofiling data on human high-grade astrocytoma to investigate the tumor-associated immune response across tumor subtypes and in relation to disease outcome in adult and pediatric astrocytoma.

## Materials and Methods

### Ethics Statement

Collection of samples used in this study was approved by the Human Research Protection Program Committee on Human Research of the University of California San Francisco. Each patient provided written consent for tissue collection, banking and research use by the UCSF Brain Tumor Research Center (BTRC).

### UCSF Genomic Profiling of Pediatric Astrocytomas

Total RNA was extracted from 24 flash-frozen pediatric astrocytoma samples described previously [24], and included 20 high-grade astrocytomas (WHO grade III and IV astrocytoma) and 4 WHO grade II diffuse astrocytomas. Total cellular RNA was extracted using Qiagen’s RNA isolation kit according to the manufacturer’s protocol, and genomic DNA contamination was removed by an on-column DNase digestion step. Expression profiling was conducted by the UCSF Sandler Center Functional Genomics Core Facility using Agilent 4×44 K arrays (G42514F). Normalization of data was performed as follows: Raw Cy3 median signal intensities were recovered from Agilent Feature Extraction software output, converted to Log_2_ scale and quantile normalized between arrays. Duplicated probe signals were merged by taking their median values. The microarray data have been deposited in the GEO database (accession number GSE38330) and described in accordance with MIAME guidelines. Unsupervised hierarchical clustering was performed using Multiple Experiment Viewer Software (MeV; http://www.tm4.org/mev/). The gene signatures identified by Paugh et al. [11] for the subtype classification of pediatric high-grade astrocytomas were applied to the UCSF pediatric astrocytoma dataset. Spearman correlation was used as the distance metric and weighted average as the linkage method.

### External Gene Expression Data Sets

Gene expression data on 173 subtyped adult GBM patients [7] (Level 3, Agilent G4502A) was obtained from the TCGA Data Portal (http://cancergenome.nih.gov) accessed on April 15, 2010. Gene expression data from the entire TCGA cohort of adult GBM patients (n = 506) as of June 16, 2011 was downloaded from the TCGA Data Portal (Level 3, Agilent G4502A). Raw gene expression data from the entire TCGA cohort of adult GBM patients (573 tumors and 10 normal brain controls as of December, 2011) was downloaded from the TCGA Data Portal (Level 1, Affymetrix HT Human Genome U133 Array). The data was pre-processed by RMA procedure [25] using custom CDF file, mapping good quality probes to unique NCBI Entrez genes [http://http://brainarray.mbni.med.umich.edu/Brainarray/Database/CustomCDF/CDF_download.asp, Version 14]. Raw gene expression data on 53 pediatric high-grade glioma patients (Affymetrix Human U133 Plus 2.0) [11] was accessed through the Gene Expression Omnibus (GEO) Website (http://www.ncbi.nlm.nih.gov/geo/, accession No. GSE19578) and was Log2 transformed and normalized using RMA.

### Gene Set Enrichment Analysis

Gene set enrichment analysis (GSEA) [26] was implemented using the Broad Institute GSEA v2.07 software (http://www.broadinstitute.org/gsea), the molecular signatures database (http://www.broadinstitute.org/gsea/msigdb), and the C5: GO gene sets database, comprised of 1454 gene sets named by GO terms and contains genes annotated by that term (www.geneontology.org). For all GSEA analyses 1000 phenotype permutations were performed and a Signal2Noise ranking metric was used to create the ranked list of genes. A false discovery rate (FDR) q-value of less than 0.25 (25%) was considered statistically significant. There was minimal overlap of genes between the subtype-specific signatures and the immune response-related gene sets: 28 of 300 genes in the adult mesenchymal signature and 29 of 236 genes in the pediatric HC3/mesenchymal signature. Gene signatures suggestive of specific immune cell subsets (**Table S1**) were also used to analyze the data. Cell-specific gene signatures of M1 and M2 polarized macrophages were compiled from analyzing multiple studies characterizing M1 and M2 macrophages [27], [28] with the majority from a profiling study of *in vitro* polarized human monocytes by Martinez et al. [28]. Hematopoietic cell lineage gene signatures (including monocytes, granulocytes, B cells, NK cells, naïve and activated T cells) were obtained from recent murine profiling [29], in which cells from peripheral blood, bone marrow or spleen were isolated and expression “fingerprints” specific to each cell type were determined. The glioma-infiltrating microglia/macrophage (GIM) gene signature was derived from differentially expressed genes (2-fold) in tumor-associated cells enriched for microglia/macrophages versus bulk tumor from a single GBM tumor [30].

### CoxBoost Prediction Modeling

CoxBoost was designed to develop proportional hazards models from microarray data and clinical covariates using a boosting approach [31]. CoxBoost modeling to identify specific immune genes associated with survival was performed in R (http://www.rprojects.org). Microarray data used to develop the model included gene expression data of 58 genes derived from the GO terms Inflammatory Response (GO:0006954) and Response to Wounding (GO:0009611). Clinical variables included age at diagnosis, Karnofsky Performance Score (KPS) and gender. Overall survival was the outcome of interest and patients alive at the time of analysis were censored. The model was created using a training set of n = 205 GBM patients from Carro et al. [9]. Variables found to be associated with overall survival at p<0.05 were verified using a permutation test and the final model was subsequently assessed via the Brier score [defined as the squared difference between an event (i.e. death) occurrence and its predicted probability] in an independent cohort of n = 301 GBM patients (TCGA Data Portal, 2011). A low Brier Score (i.e. close to 0) indicates increased predictability of the model [32].

### Immunostaining of Human Tumors

Formalin-fixed paraffin embedded tissue microarray’s (TMAs) were generated from 34 adult GBM patients and 13 pediatric astrocytoma patients who had been transcriptionally profiled. Each tumor was represented at least twice on the array. Immunohistochemistry was performed according to standard methods and immunostaining for Iba1 (019-19741 at 1∶2000, Wako Chemicals USA, Inc.), CA9 (NB100-417 at 1∶1500, Novus USA) and CD34 (CBL496 at 1∶800, Chemicon) was performed on the Ventana Medical Systems Benchmark XT. Iba1 cell counts were performed using ImageJ Software, where a minimum of three fields at 400× magnification for each tumor were counted for Iba1 positive cells and then averaged. CA9 and CD34 staining was scored in a blinded fashion in a semi-quantitative manner as follows. CA9: 0, zero positivity; 1, <10% positivity; 2, 10–25% positivity; 3, >25% positive staining. CD34: 0, density of positive blood vessels less than normal brain; 1, density equal to normal brain; 2, density greater than normal brain. Mann-Whitney Rank Sum analysis, implemented in GraphPad Prism v5.0, was used to determine differences in immunostaining between patient groups. Correlations were performed using Spearman’s rho. A test-for-trend via a linear regression model was used to evaluate a trend in Iba1 staining between subtypes in the direction of mesenchymal, classical, neural, proneural. A p-value of <0.05 was considered statistically significant.

### Differential Gene Expression Analysis and Hierarchical Clustering Based on Iba1

Iba1 scored tumors were divided into quartiles based on their microglia/macrophage cell counts and the highest quartile (high microglia/macrophages) was compared with the lowest quartile (low microglia/macrophages) for differential gene expression analysis. Significance Analysis of Microarrays [SAM [33]] was used to determine differentially expressed genes. The six genes identified as significantly differentially expressed were then applied to the whole TCGA GBM cohort (n = 573), median centered, and hierarchical clustering was performed based on these genes. Clustering analysis was performed with Spearman correlation based distance and Ward linkage.

## Results

### Adult and Pediatric High-grade Astrocytomas have Subtype-specific Enrichment of Immune Response-related Gene Sets

**Figure 1:**
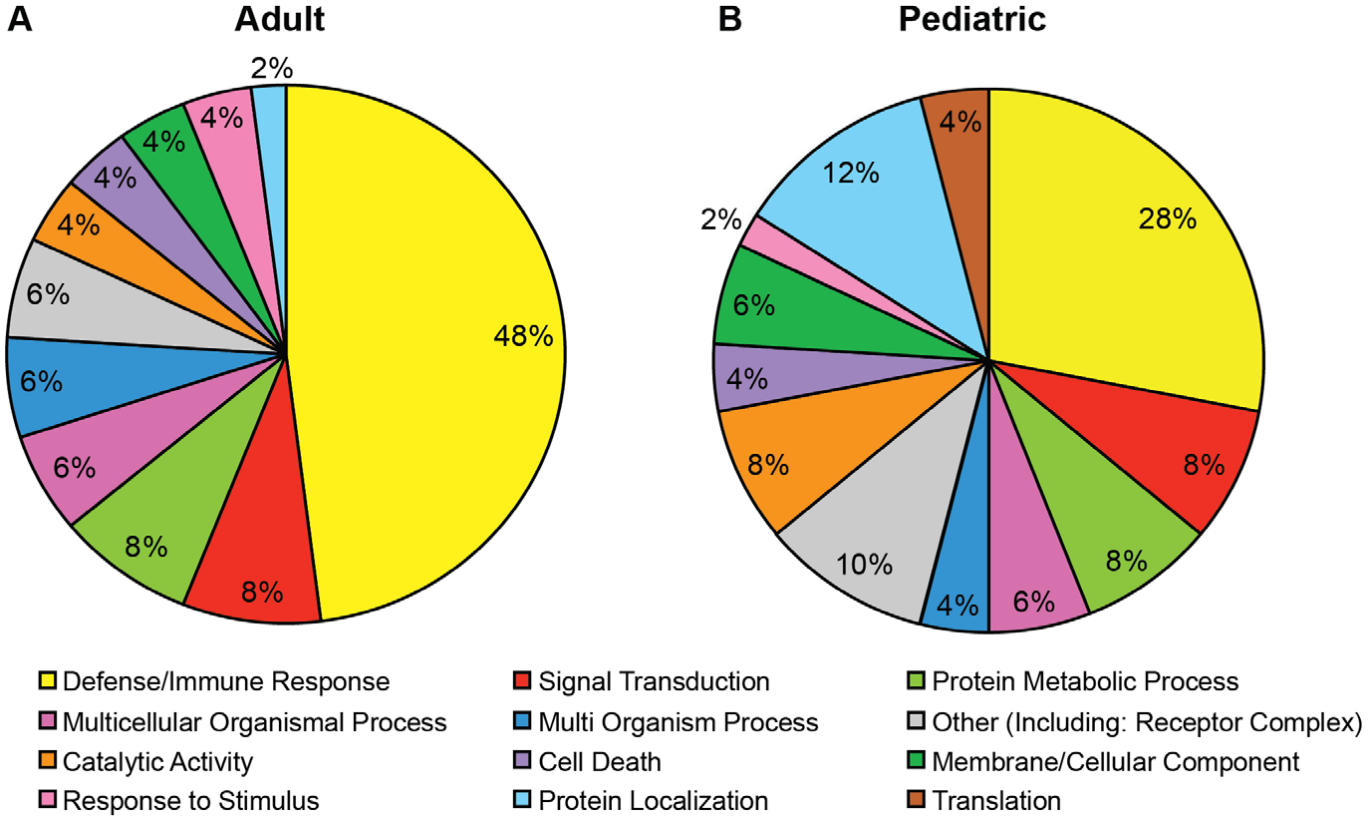
Enrichment of immune-response related gene sets in the mesenchymal subtype. GSEA analysis of 1454 gene ontology (GO) gene sets in the mesenchymal versus the non-mesenchymal subtypes in (**A**) adult GBM (TCGA, n = 173) as defined by Verhaak et al. [7] and (**B**) pediatric high-grade gliomas (grades III and IV, n = 53) as defined by Paugh et al. [11]. The top 50 enriched gene sets in the mesenchymal subtype were broadly classified by GO and are illustrated in the pie-chart as percentages. See Table S2 for complete list of top 50 enriched gene sets.

Adult GBM of the mesenchymal subtype have upregulation of genes involved in interactions with the tumor microenvironment [4], [7], [10]. To address potential relationships with immune system response in this subtype we performed gene set enrichment analysis (GSEA) on gene expression data from adult GBM previously subtyped by Verhaak et al. [7] (n = 173, TCGA Data Portal, 2010) (c5: GO gene sets). The top 50 enriched gene sets (NES: 1.74–1.89, FDR: 2.8–14%) in the mesenchymal subtype (n = 56) versus the classical, neural and proneural subtypes (n = 117) were grouped into broad GO classifications (**Figure 1A** and **Table S2A**). Strikingly, 48% of the enriched gene sets in the mesenchymal subtype were related to immune response processes, and included gene sets associated with development of the immune system, lymphocyte activation, response to infection or injury, and the adaptive immune response. Gene sets associated with signal transduction ranked second (8%) in the mesenchymal subtype and, of these, 3 of 4 were related to NF-κB signaling (**Table S2A**).

Recent gene expression profiling of pediatric high-grade gliomas (WHO grades III and IV/GBM, n = 53) [11] identified a tumor subtype (defined as HC3/mesenchymal) with a gene expression signature similar to that of the adult mesenchymal subtype proposed by Phillips et al. [4]. Similar to adult tumors, GSEA analysis of grade III and IV pediatric gliomas demonstrated enrichment of immune response-related gene sets in the HC3/mesenchymal subtype (**Figure 1B** and **Table S2B**). As the adult mesenchymal subtype proposed by Phillips et al. [4] and Verhaak et al. [7] are not identical, we used GSEA analysis to demonstrate enrichment of subtype-specific signature genes. Tumors defined as HC3/mesenchymal by Paugh et al. [11] were significantly enriched for the top 50 mesenchymal signature genes as defined by Verhaak et al. [7] (NES: 1.49, FDR: 5.4%, **Figure S1A**). Conversely, tumors defined as mesenchymal by Verhaak et al. [7] were significantly enriched for HC3/mesenchymal signature genes as defined by Paugh et al. (NES: 1.65, FDR: <0.001%, **Figure S1B**). Thereby, demonstrating similarities between the adult Verhaak mesenchymal subtype and the pediatric Paugh HC3/mesenchymal subtype.

### Validation of Tumor Subtypes and Subtype-specific Enrichment of Immune-response Genes in an Independent Cohort of Pediatric Astrocytomas

**Figure 2:**
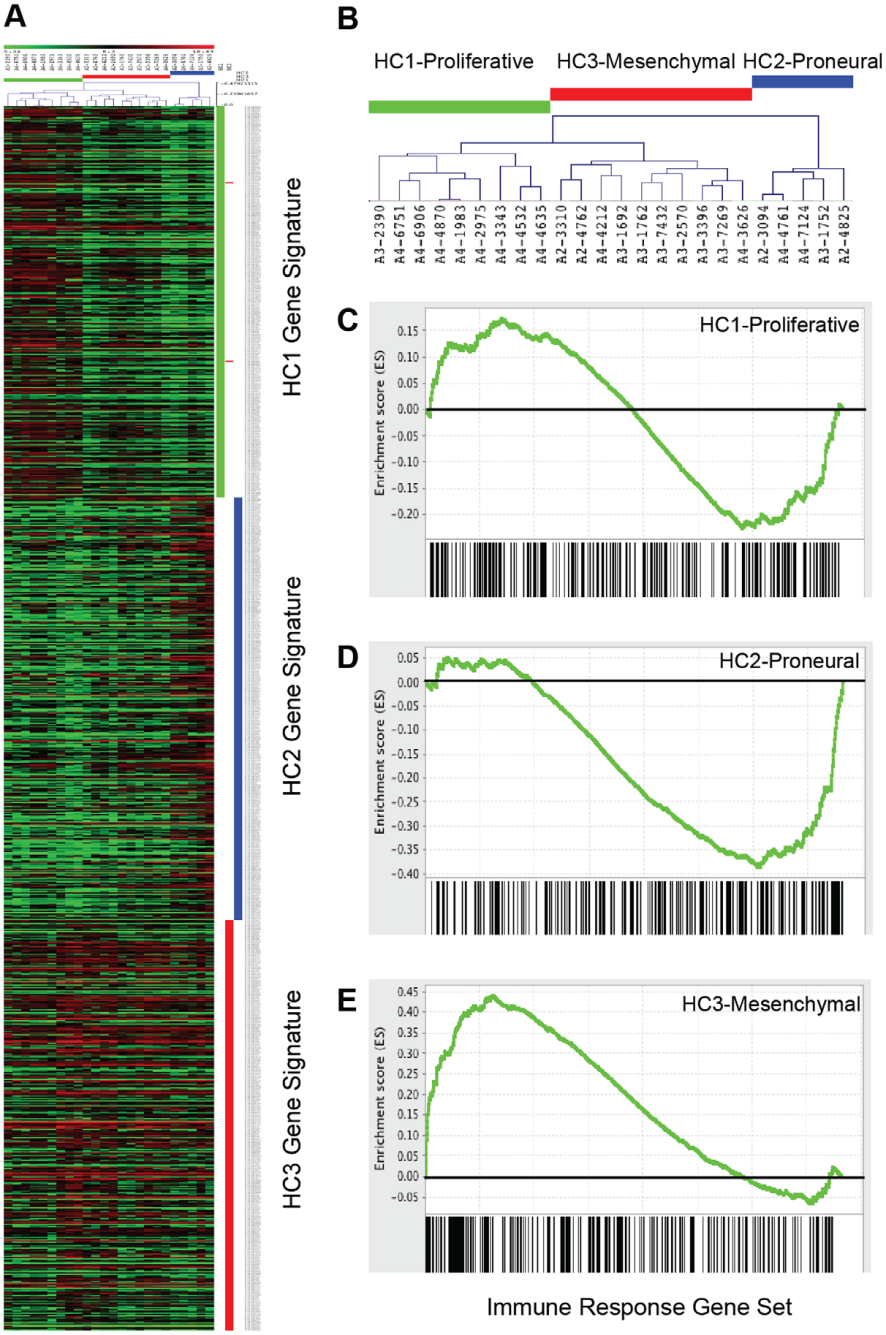
Validation of pediatric subtypes and mesenchymal enrichment of immune-response genes in an independent cohort. Pediatric astrocytomas at UCSF (grades II, III and IV, n = 24) were transcriptionally profiled and stratified using the subtype signature genes defined by Paugh et al. [11]. Unsupervised hierarchical clustering identified three major clusters resembling the previously defined subgroups: HC1/proliferative, HC2/proneural and HC3/mesenchymal. (**A**) Heatmap of clustering shows patterns of transcript expression across subtypes (red, increased expression and green, decreased expression). (**B**) Clustering dendrogram showing the three clusters/subtypes. (**C–E**) Enrichment plots showing the degree of enrichment of the “Immune Response” gene set in each of the three subgroups. (C) HC1/proliferative, NES: −0.64, FDR: 89%. (D) HC2/proneural, NES: −1.10, FDR: 34%. (E) HC3/mesenchymal, NES: 1.22, FDR: 23%.

In an independent cohort of pediatric astrocytomas from UCSF, we performed gene expression profiling (grades II, III and IV, n = 24), applied 1035 signature genes proposed by Paugh et al. [11] to define tumor subtypes, and performed unsupervised hierarchical clustering. Three major clusters representative of the three previously defined subtypes were identified: HC1/proliferative, HC2/proneural, HC3/mesenchymal (**Figure 2A–B**). In the UCSF pediatric HC3/mesenchymal cohort, immune response GO gene sets were also significantly enriched (**Table S3**). **Figure 2C, D, E** demonstrates selective enrichment of the Immune Response gene set (GO:0006955) in the HC3/mesenchymal group (NES: 1.22, FDR: 23.7%), further supporting an association between the mesenchymal subtype and increased expression of immune response-related genes.

### Microglia/macrophage Gene Signatures Associated with the Mesenchymal Subtype in GBM

**Figure 3:**
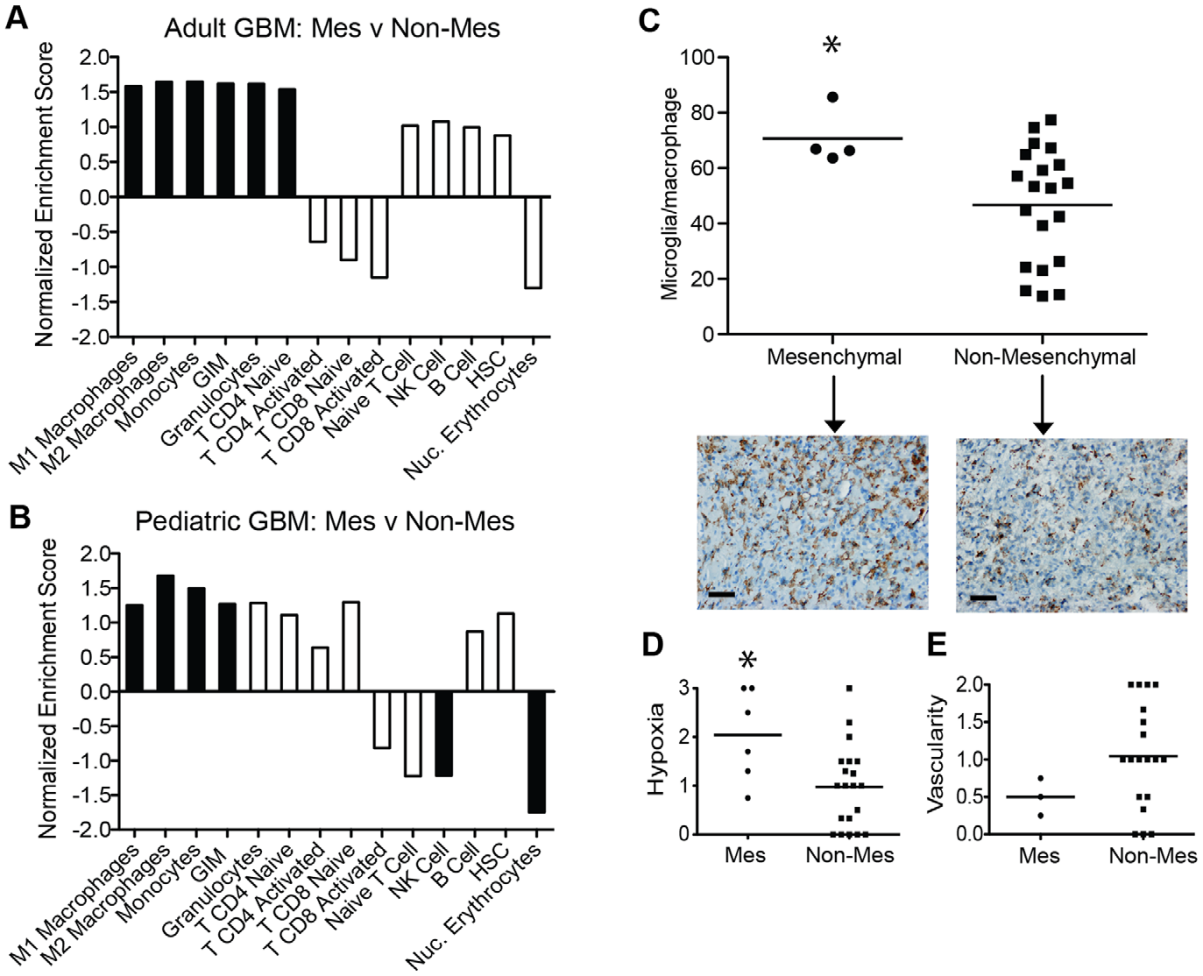
Enrichment of microglia/macrophage gene signatures in the mesenchymal subtype. Immune cell-specific gene expression signatures were used to suggest dominant inflammatory cell populations across tumor subtypes. GSEA analysis comparing the mesenchymal (Mes) to non-mesenchymal (Non-Mes) subtypes was performed in (**A**) adult GBM (TCGA, n = 173) and (**B**) pediatric GBM (grade IV, n = 38) [11]. Bars show the normalized enrichment score for each cell-specific gene signature. Black bars represent gene signatures with significant enrichment (FDR<25%), white bars represent gene signatures which did not reach significance (FDR>25%). GIM: glioma-infiltrating microglia/macrophages, HSC: hematopoietic stem cells. See Table S1 for signature gene lists and Table S4 for complete GSEA data output. (**C**) Numbers of microglia/macrophages per 400× field, as determined by immunostaining for Iba1, across tumor subtypes in adult GBM (n = 24). Each dot represents an individual patient tumor and the mean is denoted by the line. Microglia/macrophage cell numbers are significantly higher in the mesenchymal subtype compared to the non-mesenchymal subtype (* Mann-Whitney, p = 0.04). Representative images of Iba1 staining for the mesenchymal and non-mesenchymal subtypes are shown (Iba1 cell counts of 66.8 and 44.6, respectively), scale bars = 50 µm. (**D–E**) The extent of (D) hypoxia, as determined by CA9 immunostaining (n = 22), and (E) vascularity, as determined by CD34 (n = 26), was compared across tumor subtypes in adult GBM. Each dot represents an individual patient tumor and the mean is denoted by the line. The level of hypoxia is significantly higher in the mesenchymal compared to non-mesenchymal subtype (* Mann-Whitney, p = 0.027), however vascularity was not significantly different (Mann-Whitney, p = 0.173).

Having demonstrated an upregulation of immune response-related genes in the mesenchymal subtype we aimed to characterize the cell types potentially driving this pattern of gene expression. Immune cell-specific gene signatures (**Table S1**) were identified from recent profiling studies [27]–[30] and utilized for GSEA analysis. In adult GBM, six of 14 cell-specific gene signatures, were enriched in the mesenchymal subtype (NES: 1.53–1.64, FDR: 1.5–3.2%) (**Figure 3A** and **Table S4A**). The six enriched gene sets included gene signatures associated with microglia/macrophages, M1 and M2 macrophages and glioma infiltrating microglia/macrophages (GIM), along with the monocyte, granulocyte and naïve CD4 T cell gene sets. Similar to adults, the HC3/mesenchymal subtype of pediatric GBM (grade IV, n = 38) demonstrated significant enrichment of the gene signatures associated with microglia/macrophages and monocytes (NES: 1.25–1.67, FDR: 1.6–24%) (**Figure 3B** and ****). Unlike adults, the gene sets representing granulocytes and naïve CD4 T cells were not significantly enriched and two gene sets were negatively enriched; NK cells and nucleated erythrocytes (NES: −1.21 and −1.74, FDR: 22% and 0.1%, respectively). A similar analysis was performed with the independent UCSF cohort of pediatric astrocytomas. Due to the small number of GBMs (n = 2) in the HC3/mesenchymal subtype (**Figure S2**), we included all astrocytoma grades (II, III and IV) in the analysis. Similar to the larger pediatric GBM data set, there was significant enrichment of the M2 macrophage gene signature (NES: 1.5, FDR: 18%) and significant negative enrichment of nucleated erythrocytes (NES: −1.49, FDR: 9.7%), in the HC3/mesenchymal subtype (**Figure S3A and Table S4C**).

### Microglia/macrophage Infiltrate in Adult Mesenchymal GBM

Increased expression of microglia/macrophage-related genes could reflect increased numbers of immune cells or increased transcript expression levels per cell. To determine whether the mesenchymal subtype contained a greater number of microglia/macrophages, we generated tissue microarrays from adult subtyped GBM (n = 24) and from UCSF pediatric subtyped astrocytomas (n = 13), and analyzed microglia/macrophage number, as determined by Iba1 immunohistochemistry. While there was inter-tumoral heterogeneity, the mean microglia/macrophage number was significantly greater in the mesenchymal subtype of adult GBMs than in the non-mesenchymal subtype (Mann Whitney p = 0.04, **Figure 3C**). Comparing across the four tumor subtypes there was a linear trend of decreasing numbers of microglia/macrophages in the order of mesenchymal, classical, neural and proneural (test-for-trend p = 0.02, **Figure S4A**). Microglia/macrophage recruitment can be regulated via multiple mechanisms including levels of HIF1α [16]. Interestingly, hypoxia, as determined by CA9, was significantly higher in tumors of the mesenchymal versus the non-mesenchymal subtype, (Mann-Whitney p = 0.027, **Figure 3D**). While both immune cell recruitment and HIF1α can promote angiogenesis [16], we did not identify a significant difference in vascularity, as determined by CD34 immunostaining, between tumors of the mesenchymal and non-mesenchymal subtype (Mann-Whitney p = 0.173, **Figure 3E**). In the UCSF pediatric cohort the tumors represented a diversity of grades (grades II, III and IV) and microglia/macrophage number did not differ between mesenchymal and non-mesenchymal tumors (Mann Whitney p = 0.414, **Figure S3B**). Subtype specific comparisons are shown in **Figure S4**.

### Association of Immune Response-related Gene Expression and Survival

Previous studies have identified a gene signature associated with worse prognosis in adult GBM [9]. To examine whether immune response-related genes are associated with survival, independent of tumor subtype, we performed Gene Ontology (GO) analysis of this signature gene set and 2 of the top 3 GO terms were immune response-related (“Inflammatory Response” and “Response to Wounding”, including a total of 58 independent genes, **Table S5**). To identify the immune response-related genes in this list of 58 that are most closely associated with survival in a robust and multivariate manner, we used CoxBoost modeling [31]. The training set was comprised of gene expression data from 205 adult GBM previously used to define the worst prognosis signature [9] and the independent validation set was comprised of data from 301 independent GBM tumors (TCGA Data Portal, 2011). Clinical variables were included in the model, and as expected, age and KPS score were found to be significantly associated with survival (p<0.005, **Table 1**). In addition, the expression of 12 potential immune response genes were also significantly associated with overall survival: SPP1, PDPN, IL1RN, IL6, CCL8, MDK, IL1RAP, IRF7, MASP2, CASP3, TFRC and HRH4 (**Table 1**). Validation of the model in the independent data set resulted in a low Brier Score of 0.0793, demonstrating the strength of the model and the association between immune response gene expression and poor prognosis.

**Table 1:** Immune response-related genes most strongly associated with survival in adult GBM^a^.

	Name [entrez]	p-value	Coefficient^b^	Hazard Ratio
Age	age at diagnosis (in years)	<0.005	0.2934	1.3411
SPP1	secreted phosphoprotein 1 [6696]	<0.005	0.1256	1.1339
PDPN	podoplanin [10630]	<0.005	0.1049	1.1107
IL1RN	interleukin 1 receptor antagonist [3557]	<0.005	0.0310	1.0315
IL6	interleukin 6 [3569]	<0.005	0.0150	1.0152
KPS	karnofsky performance score	<0.005	−0.1541	0.8571
CCL8	chemokine (C-C motif) ligand 8 [6355]	0.0016	0.0804	1.0838
MDK	midkine [4192]	0.0016	0.0665	1.0689
IL1RAP	interleukin 1 receptor accessory protein [3556]	0.0016	0.0543	1.0558
IRF7	interferon regulatory factor 7 [3665]	0.0016	0.0532	1.0547
MASP2	mannan-binding lectin serine peptidase 2 [10747]	0.0049	0.1415	1.1521
CASP3	caspase 3 [836]	0.0131	0.0583	1.0601
TFRC	transferrin receptor [7037]	0.0229	0.0248	1.0252
HRH4	histamine receptor H4 [59340]	0.0426	0.0892	1.0933
Gender	patient gender (female is baseline)	0.0655	−0.0786	0.9243

a CoxBoost modeling was performed on 58 immune response-related genes from the TCGA worst prognosis signature [9].

b Coefficient for the standardized covariates in the Cox proportional hazards model.

To assess if these survival-related immune response genes may be expressed by the microglia/macrophage population within the tumor mass we examined gene expression profiling data from tumor-associated cells enriched for microglia/macrophages compared with bulk tumor from a single GBM sample [30]. As shown in **Figure S5**, eight of these immune response genes (SPP1, PDPN, IL1RN, IL6, CCL8, MDK, IRF7, and HRH4) were expressed between 1.25–1.59 fold higher in the microglia/macrophage enriched population compared to bulk tumor. This fold change was comparable to the microglia/macrophage specific markers CD45, CD11b and Iba1 (1.28–1.69 fold).

### Enrichment of Microglia/macrophage-specific Gene Sets in Poor Prognosis Adult GBM

**Figure 4:**
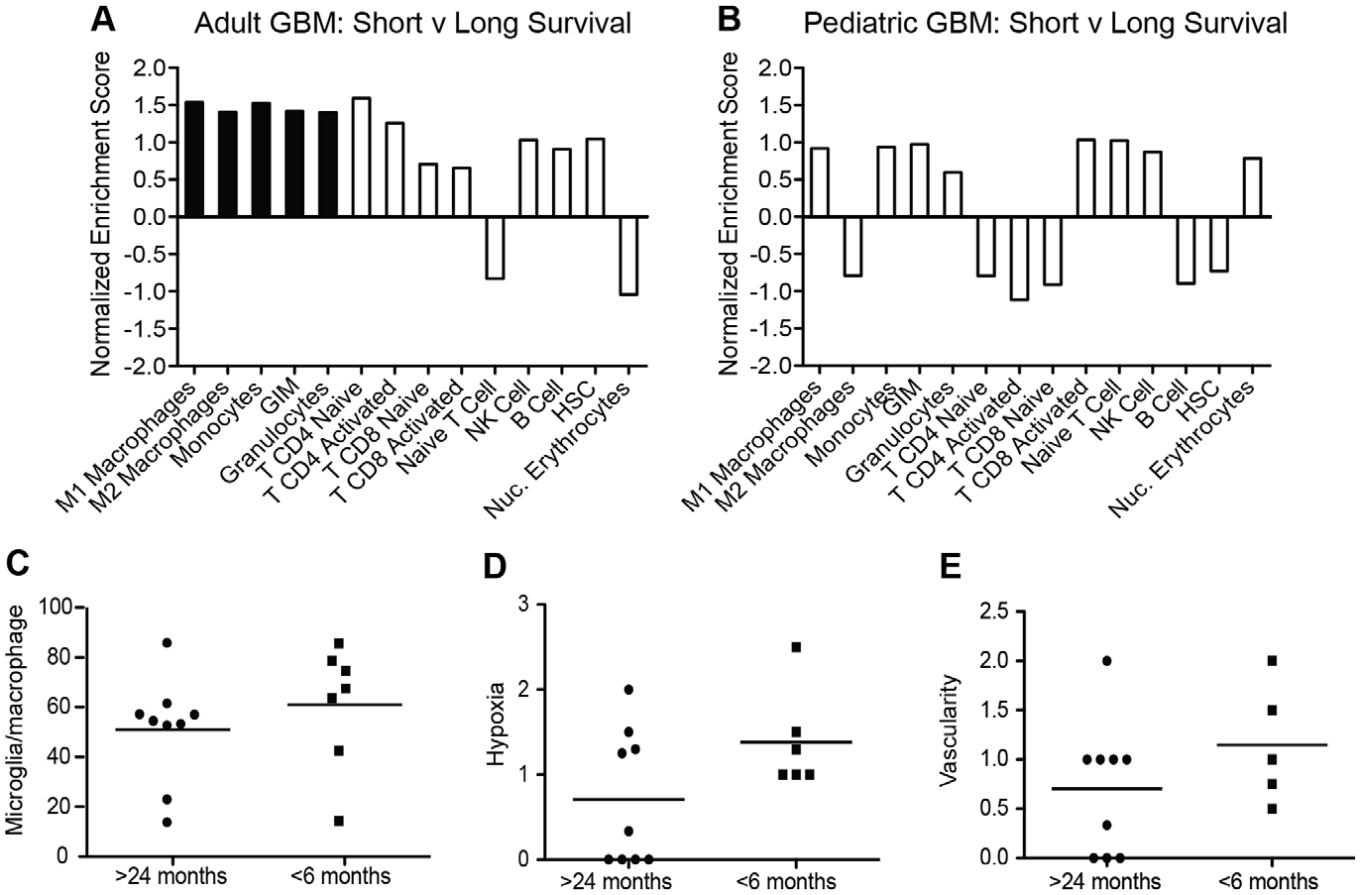
Enrichment of microglia/macrophage gene signature in adult GBM with short survival. (**A**) Adult GBM patients (TCGA, n = 506) and (**B**) pediatric GBM patients (grade IV, n = 38) were grouped according to their survival times. Tumor gene expression was analyzed for enrichment of cell-specific gene signatures in short survival (<6 months) versus long survival (>24 months) patients. Bars denote the normalized enrichment score for each cell-specific gene signature. Black bars represent gene signatures with significant enrichment (FDR<25%), white bars are those which did not reach significance (FDR>25%). GIM: glioma-infiltrating microglia/macrophages, HSC: hematopoietic stem cells. See Table S6 for complete GSEA data output. (**C–E**) Analysis of (C) microglia/macrophage cell numbers per 400× field (Iba1), (D) hypoxia (CA9) and (E) vascularity (CD34) in adult GBM patients with short (<6 months, n = 6) and long (>24 months, n = 9) survival. Each dot represents an individual patient tumor and the mean is denoted by the line.

Rather than stratify tumors by subtype, adult GBM patients (n = 506) were stratified based on survival (short survival, <6 months, n = 98 and long survival, >24 months, n = 78), and GSEA analysis demonstrated an enrichment of all macrophage, monocyte, and granulocyte gene signatures in short survival patients (NES: 1.39–1.53, FDR: 15–21%) (**Figure 4A** and **Table S6A**). In contrast to adult GBM, a similar stratification of pediatric GBM into short (<6 months, n = 9) and long (>24 months, n = 12) survival groups did not reveal a significant enrichment of immune cell-specific gene signatures in the short survival group (**Figure 4B** and **Table S6B**).

In the subset of adult GBM for which tissue was available for analysis, there was not a significant increase in mean number of microglia/macrophages in tumors from patients with shorter survival (p = 0.252, **Figure 4C**). Interestingly, there was also no significant increase in hypoxia (p = 0.209, **Figure 4D**) or vascularity (p = 0.303, **Figure 4E**).

### Stratification of Adult GBM Patients by Microglia/macrophage Infiltrate

**Figure 5:**
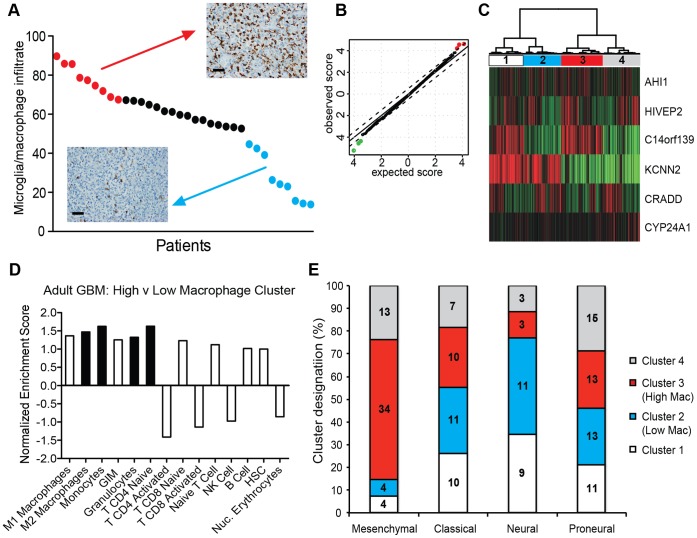
Determination of gene expression signature for high microglia/macrophage infiltrate and association with cell-specific gene signatures and molecular subtypes. (**A**) Variation in number of tumor-associated microglia/macrophage in 34 GBM tumors. Each dot represents an individual patient tumor, red denotes tumors defined to have high numbers of microglia/macrophages (greater than 67.5 microglia/macrophages per 400× field), blue dots denote tumors defined to have low microglia/macrophage numbers (less than 44.6 microglia/macrophages per 400× field). A representative image of microglia/macrophage staining (Iba1) for each group is shown (scale bars = 50 µm). (**B**) Q-Q plot of genes differentially expressed in high microglia/macrophage tumors (n = 9) and low microglia/macrophage tumors (n = 9), as denoted in (A). (**C**) Unsupervised hierarchical clustering of adult GBMs (TCGA, n = 573), based on the six differentially expressed genes between high and low microglia/macrophage tumors as denoted in (A) and shown in Table 2, identified four distinct clusters (red, increased expression and green, decreased expression). Low microglia/macrophage tumors were primarily in Cluster 2 (6/9) and high microglia/macrophage tumors were predominantly in Cluster 3 (8/9). (**D**) Enrichment of cell-specific gene signatures was analyzed in Cluster 3 tumors (surrogate high microglia/macrophage) as compared to Cluster 2 tumors (surrogate low microglia/macrophage). Bars denote normalized enrichment score for each cell-specific gene signature. Black bars represent gene signatures with significant enrichment (FDR<25%), white bars are those which did not reach significance (FDR>25%). GIM: glioma-infiltrating microglia/macrophages, HSC: hematopoietic stem cells. See Table S7 for complete GSEA data output. (**E**) Distribution of molecular subtypes across microglia/macrophage signature clusters. The numbers within the bars express the number of tumors within each subtype/cluster combination.

To identify factors associated with increased microglia/macrophage infiltrates, we stratified adult tumors (n = 34) by number of tumor-associated microglia/macrophages (Iba1) and analyzed them by immunohistochemistry and differential gene expression analysis. Ignoring tumor subtype, there was no significant association between microglia/macrophage number and levels of hypoxia or vascularity in our cohort of adult GBM (p = 0.674 and p = 0.987 respectively, **Figure S6**). Comparing tumors with the highest and the lowest number of microglia/macrophages (top 25%, n = 9 vs bottom 25%, n = 9, **Figure 5A**) we identified 3 genes significantly upregulated and 3 genes significantly downregulated (**Figure 5B** and **Table 2**). Using these six genes as a surrogate marker for microglia/macrophage infiltrate, we identified four distinct tumor clusters based on unsupervised hierarchical clustering of gene expression data (n = 573 tumors and n = 10 normal samples, TCGA; **Figure 5C** **)**. Eight of nine high microglia/macrophage tumors were in Cluster 3 and six of nine low microglia/macrophage tumors were in Cluster 2.

**Table 2:** Genes differentially expressed in tumors with high versus low numbers of microglia/macrophage.

		Mean transcript expression^a^		
Gene symbol	Gene name [Entrez]	High	Low	p-value
Upregulated Genes				
HIVEP2	*human immunodeficiency virus type I enhancer binding protein 2 [3097]*	6.29	5.33	0.00011
C14orf139	*long intergenic non-protein coding RNA 341 [79686]*	7.26	5.75	0.00014
AHI1	*abelson helper integration site 1 [54806]*	5.08	4.59	0.00021
Downregulated Genes				
CYP24A1	*cytochrome P450, family 24, subfamily A, polypeptide 1 [1591]*	3.31	3.61	3E-6
KCNN2	*potassium intermediate/small conductance calcium-activated channel,* *subfamily N, member 2 [3781]*	6.08	8.17	0.00004
CRADD	*CASP2 and RIPK1 domain containing adaptor with death domain [8738]*	6.50	7.3	0.00043

a Mean gene expression (Log_2_) for microglia/macrophage high (n = 9) and low tumors (n = 9) in adult GBM.

GSEA analysis of Cluster 3 (surrogate high microglia/macrophage, n = 150) versus Cluster 2 (surrogate low microglia/macrophages, n = 129) demonstrated significant enrichment of the M2 macrophage and monocyte gene signatures (NES: 1.47 and 1.62, FDR: 18% and 9.9%, respectively) (**Figure 5D** and **Table S7**), validating our immune cell type specific gene signatures. Furthermore, Cluster 3 had significant enrichment of the granulocyte and CD4 naïve T cell signatures (NES: 1.32 and 1.62, FDR: 24% and 19%, respectively), suggesting a potential association between high microglia/macrophage infiltrate and increased expression of characteristic granulocyte and CD4 T cell genes.

Protein analysis demonstrated tumors of the mesenchymal subtype exhibited high microglia/macrophage numbers in contrast to non-mesenchymal tumors that had prominent inter-tumoral heterogeneity of microglia/macrophage number (**Figure 3**). Using our high microglia/macrophage gene signature, tumors in Cluster 3 were enriched for tumors of the mesenchymal subtype, with 62% (34/55) of mesenchymal tumors falling into Cluster 3 (**Figure 5E** **)**. Consistent with heterogeneity within tumor subtypes, Cluster 3 also contained non-mesenchymal tumors, including classical, neural and proneural tumors (**Figure 5E**). There was no significant difference in survival between patients in Cluster 3 versus Cluster 2 as a group or when analyzed within individual subtypes (data not shown).

## Discussion

In this study we analyzed primary human astrocytomas to investigate immune response indicators across tumor subtypes, and in relation to disease outcome. We demonstrate a striking enrichment of immune response-related genes and genes characteristic of myeloid cells including microglia, macrophages, and monocytes in the mesenchymal subtype of adult and pediatric GBM. Importantly, in adult GBMs, microglia/macrophage, monocyte and granulocyte cell signatures were significantly enriched in tumors from patients with the shortest survival. Interestingly, this was not the case for pediatric GBM: i.e., immune cell-specific signature genes were not enriched in children with the shortest survival. In adults, tumors having a gene expression signature associated with high microglia/macrophage numbers exhibited enhanced expression of characteristic myeloid, granulocyte and CD4 T cell genes and were enriched for tumors of the mesenchymal subtype. These data support marked inter-tumoral differences in the microglia/macrophage response.

Molecular stratification of adult GBM into subtypes has provided a framework for investigations into differential disease pathogenesis and tumor response to therapy [3]–[7]. However, to date, tumor profiling of GBM has concentrated on analysis of bulk tumor with little attention directed to the non-neoplastic elements, including immune cells, present in the tumor. In the current study we examined the pattern of gene expression signatures characteristic of specific immune cell populations. In adult GBM, immune response genes, including genes representative of microglia/macrophage populations, were enriched in tumors of the mesenchymal subtype. This pattern of gene expression was consistent with the increased numbers of microglia/macrophages identified in mesenchymal as compared to non-mesenchymal GBMs. Furthermore, we identified enrichment of microglia/macrophage-related signature genes in patients with the shortest survival. We suggest microglia/macrophage function may be particularly pronounced in a subset of GBM with aggressive clinical behavior. Further characterization of this subset may be particularly important for identifying patients for clinical trials involving immunomodulatory therapies. Indeed, a recent study of dendritic cell vaccination demonstrated improved responses in patients of the mesenchymal subtype compared to other subtypes [34]. PLX3397, an inhibitor of macrophage colony stimulating factor 1, and other such therapies aimed at inhibiting the microglia/macrophage response, may also prove particularly effective in this molecularly defined subset of GBM.

Our data indicate an enrichment of microglia/macrophage-related signature genes in tumors from patients with short survival. As increased gene expression may reflect both differences in cell number and differences in microglia/macrophage phenotype or activation state, we examined microglia/macrophage number in available UCSF tumors. Increased absolute numbers of microglia/macrophages was not associated with short survival in this GBM cohort. This discrepancy with the gene expression data may be a result of intra-tumoral heterogeneity, alternatively it may suggest possible differences in gene expression levels per cell. To identify immune response-related genes most strongly associated with survival we performed CoxBoost modeling on immune response genes identified in a worse prognosis gene signature of adult GBM [9]. In addition to established clinical variables, 12 potential immune response-related genes were found to be significantly associated with poor survival. These included genes known to be expressed by microglia (CCL8 [35], SPP1 [36], IRF7 [37], IL1RAP [38]), macrophages (IL1RN [39], SPP1 [40], PDPN [41], IL1RAP [42], MDK [43], TFRC [44], HRH4 [45]), and tumor-associated macrophages (IL6 [46], SPP1 [47]). Many of the corresponding gene products are also known to influence monocyte/microglia/macrophage recruitment and activation (IL6 [48], SPP1 [49], CCL8 [50], MDK [51]).

Some of the genes identified by CoxBoost modeling have been previously associated with GBM disease outcome. Notably, PDPN was included in a 9 gene prognostic signature defined by Colman et al. [6], the inflammatory cytokine IL6 has been linked to poor prognosis in GBM [52] and high serum levels of the secreted glycoprotein SPP1 (osteopontin) correlate with poor survival in GBM [53]. Our studies, similar to all studies that analyze expression data from bulk tumor, are limited by the inability to definitively identify the cellular source of a particular transcript. However, analysis of gene expression data from purified human tumor-associated microglia/macrophages demonstrated increased expression of some of these genes (8/12) relative to bulk tumor. Thus total expression levels of many of these survival-related immune genes is likely derived in part from the microglia/macrophage infiltrate.

To elucidate other factors associated with a high microglia/macrophage infiltrate, adult tumors were stratified by microglia/macrophage number into high and low subsets and based on the differentially expressed genes, hierarchical clustering was performed on a large set of profiled GBMs. Tumors with a high microglia/macrophage gene expression signature demonstrated specific enrichment of macrophage, granulocyte and CD4 T cell gene sets. In mammary adenocarcinoma, a significant interplay between CD4+ T cells and macrophages in promoting invasion and metastasis has been demonstrated [54], and it is possible that such interactions may also occur in a subset of GBM. While tumors with a high microglia/macrophage gene expression signature were most commonly tumors of the mesenchymal subtype they also included other tumor subtypes suggesting potential biologic differences within the tumor subtypes.

The gene expression signature associated with high microglia/macrophage number was most strongly driven by two genes: KCNN2 and C14orf139. KCNN2 or SK2, a calcium activated potassium channel, is induced in activated microglia and helps to regulate the microglia respiratory burst function [55]. KCNN2 expression has been previously reported in human glioma [56], and in melanoma KCNN2 is thought to help regulate hypoxia-induced cell proliferation [57]. The function of C14orf139 is currently unknown.

While there exist some similarities between pediatric and adult high-grade astrocytomas, a substantial literature attests to anatomic, histologic, and molecular differences in adult versus pediatric disease [2], [11]. Our data suggest the immune response may represent one of these differences. Recently, pediatric high-grade gliomas have been divided into three subtypes based on patterns of gene expression. While the pediatric HC3/mesenchymal subtype-specific signature genes are not identical to those used for adult tumors [4], [7], they share common elements with the adult mesenchymal subtype of Phillips et al. [4], [11] and we demonstrate similarities with the mesenchymal subtype of Verhaak et al. [7]. Using these signature genes [11] we were able to stratify an independent cohort of pediatric grade II, III and IV astrocytomas into the three defined subtypes. To our knowledge, this is the first study validating the stratification of pediatric astrocytomas into transcriptionally defined classes. These data emphasize the robustness of the gene signatures and subtypes defined by Paugh et al. [11].

Similar to adult tumors, the pediatric HC3/mesenchymal subtype was enriched in immune response-related genes in both the UCSF and Paugh et al. [11] pediatric cohorts. Furthermore, pediatric HC3/mesenchymal GBM were also enriched for gene signatures of microglia/macrophages and monocytes, emphasizing a potential subtype-specific role for these immune cells in adult and pediatric tumors. In contrast to adult GBM, the microglia/macrophage gene signatures were not significantly enriched in tumors from short survival patients among the pediatric GBM cohort. While these results must be interpreted with caution given the relatively small number of pediatric tumors analyzed, it emphasizes potentially important differences in tumor biology between adult and pediatric patients.

Taken together, our study demonstrates increased expression of immune response related genes, including microglia/macrophage signature genes, in a subset of adult and pediatric GBM. Understanding the factors that drive this differential immune response and its implications for therapeutic decision-making is critical. Future studies will be designed to elucidate these differences.

## Supporting Information

Figure S1 **Comparison of gene expression across different mesenchymal tumor subtypes.**
(PDF)

Figure S2 **Distribution of tumor grade between tumor subtypes in two pediatric astrocytoma cohorts.**
(PDF)

Figure S3 **GSEA analysis of cell-specific gene signatures and number of microglia/macrophages in mesenchymal versus non-mesenchymal subtypes in the UCSF pediatric astrocytoma cohort.**
(PDF)

Figure S4 **Subtype specific comparisons of microglia/macrophage cell number, hypoxia and vascularity in adult and pediatric astrocytomas.**
(PDF)

Figure S5 **Expression of survival-associated immune response-related genes in glioma infiltrating microglia/macrophages relative to bulk tumor.**
(PDF)

Figure S6 **Correlation between microglia/macrophage cell number and hypoxia or vascularity in adult GBM.**
(PDF)

Table S1 **Cell-specific gene signatures used for GSEA analysis.**
(XLSX)

Table S2 **Enrichment scores and statistics of the top 50 enriched GO gene sets from GSEA analysis of the mesenchymal versus non-mesenchymal adult and pediatric astrocytoma subtypes.**
(XLSX)

Table S3 **Enrichment scores and statistics of defense/immune response GO gene sets from GSEA analysis of the mesenchymal versus non-mesenchymal subtypes in the UCSF pediatric astrocytoma cohort.**
(XLSX)

Table S4 **Enrichment scores and statistics of cell-specific gene signatures from GSEA analysis of the mesenchymal versus non-mesenchymal subtypes in adult and pediatric astrocytomas.**
(XLSX)

Table S5 **Gene Ontology (Biological Process) analysis of the TCGA Worst Prognosis Signature.**
(XLSX)

Table S6 **Enrichment scores and statistics of cell-specific gene signatures from GSEA analysis of short versus long survival patients in adult and pediatric astrocytomas.**
(XLSX)

Table S7 **Enrichment scores and statistics of cell-specific gene signatures from GSEA analysis of Cluster 3 (surrogate high microglia/macrophage) versus Cluster 2 (surrogate low microglia/macrophage) in adult GBM.**
(XLSX)
