# Supplementary material for: Increased Microglia/Macrophage Gene Expression in a Subset of Adult and Pediatric Astrocytomas
Source: PLoS One. 2012 Aug 22;7(8):e43339. doi: 10.1371/journal.pone.0043339 (PMC3425586; doi:10.1371/journal.pone.0043339)
Supplement: Figure S1 — Comparison of gene expression across different mesenchymal tumor subtypes. (PDF) [file pone.0043339.s001.pdf]

Engler et al, 2012, PLoS ONE

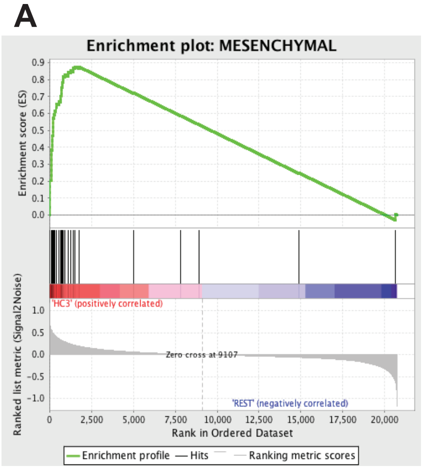

Figure S1. Comparison of gene expression across different mesenchymal tumor subtypes. (A) Significant enrichment of the top 50 genes from the Verhaak [7] mesenchymal signature in the Paugh [11] HC3/mesenchymal subtype. **(B)** Significant enrichment of the Paugh HC3/mesenchymal signature in the Verhaak mesenchymal subtype.
