# Supplementary material for: Increased Microglia/Macrophage Gene Expression in a Subset of Adult and Pediatric Astrocytomas
Source: PLoS One. 2012 Aug 22;7(8):e43339. doi: 10.1371/journal.pone.0043339 (PMC3425586; doi:10.1371/journal.pone.0043339)
Supplement: Figure S2 — Distribution of tumor grade between tumor subtypes in two pediatric astrocytoma cohorts. (PDF) [file pone.0043339.s002.pdf]

Engler et al, 2012,PLoS ONE

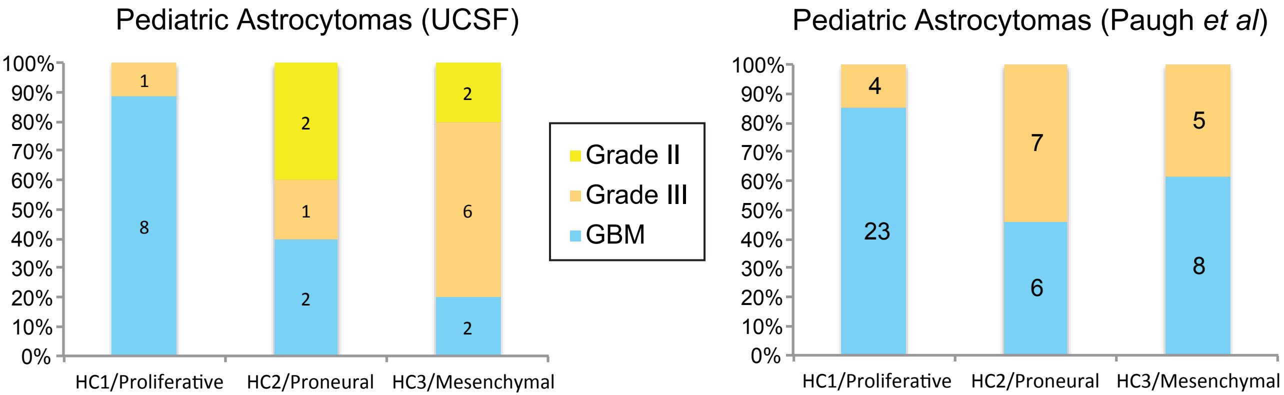


**Figure S2. Distribution of tumor grade between tumor subtypes in two pediatric astrocytoma cohorts.** The HC3/mesenchymal subgroup is predominantly Grade IV/GBM (61%) in the Paugh *et al* [11] cohort while it is more heterogeneous in the UCSF cohort with only 20% GBM.
