# Supplementary material for: Increased Microglia/Macrophage Gene Expression in a Subset of Adult and Pediatric Astrocytomas
Source: PLoS One. 2012 Aug 22;7(8):e43339. doi: 10.1371/journal.pone.0043339 (PMC3425586; doi:10.1371/journal.pone.0043339)
Supplement: Figure S3 — GSEA analysis of cell-specific gene signatures and number of microglia/macrophages in mesenchymal versus non-mesenchymal subtypes in the UCSF pediatric astrocytoma cohort. (PDF) [file pone.0043339.s003.pdf]

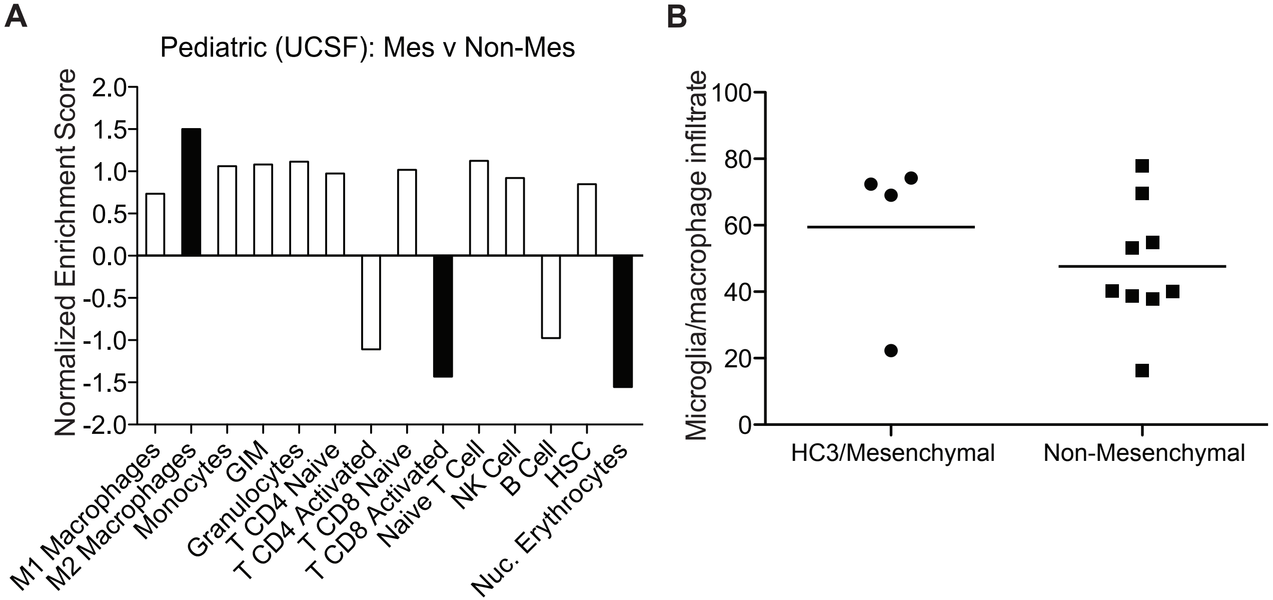

Figure S3. GSEA analysis of cell-specific gene signatures and number of microglia/macrophages in mesenchymal versus non-mesenchymal subtypes in the UCSF pediatric astrocytoma cohort. (A) GSEA analysis comparing the mesenchymal subtype (Mes, n=10) to all other subtypes (Non-Mes, n=14) in the UCSF cohort of pediatric astrocytomas (grades II, III and IV). Bars show the normalized enrichment score for each cell-specific gene signature. Black bars represent gene signatures with significant enrichment (FDR<25%), white bars are those which did not reach significance (FDR>25%). GIM: glioma-infiltrating microglia/macrophages, HSC: hematopoietic stem cells. See Table S4C for complete data. **(B)** The tumor-associated microglia/macrophage infiltrate, as determined by immunohistochemistry for Iba1, was compared across tumor subtypes in the UCSF cohort of pediatric astrocytomas (n=13). Each dot represents an individual patient tumor and the mean is shown by the line. No difference in Iba1 staining was seen between mesenchymal and non-mesenchymal tumors (Mann Whitney, p=0.414).
