# Supplementary material for: Increased Microglia/Macrophage Gene Expression in a Subset of Adult and Pediatric Astrocytomas
Source: PLoS One. 2012 Aug 22;7(8):e43339. doi: 10.1371/journal.pone.0043339 (PMC3425586; doi:10.1371/journal.pone.0043339)
Supplement: Figure S4 — Subtype specific comparisons of microglia/macrophage cell number, hypoxia and vascularity in adult and pediatric astrocytomas. (PDF) [file pone.0043339.s004.pdf]

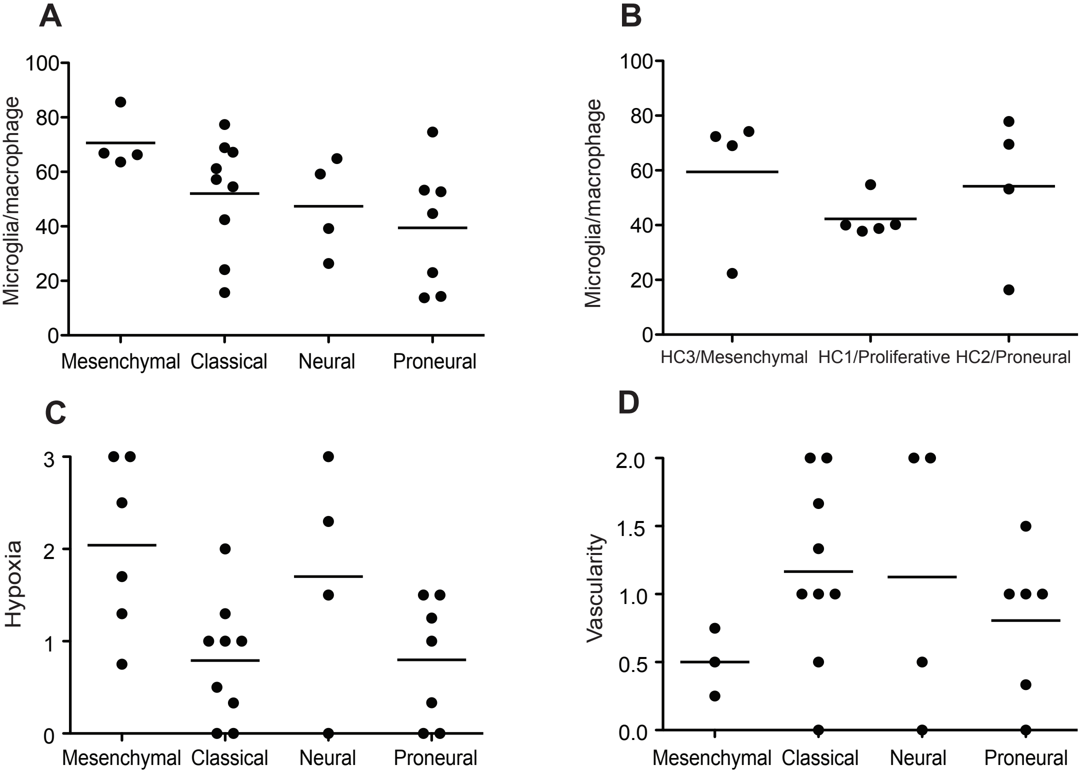

Figure S4. Subtype specific comparisons of microglia/macrophage cell number, hypoxia and vascularity in adult and pediatric astrocytomas. Subtype specific comparisons of **(A)** migroglia/macrophage cell numbers per 400x field (Iba1 immunostaining) in adult GBM and **(B)** pediatric astrocytoma (grades II, III and IV), **(C)** hypoxia (CA9 immunostaining) in adult GBM and **(D)** vascularity (CD34 immunostaining) in adult GBM.
