# Supplementary material for: Increased Microglia/Macrophage Gene Expression in a Subset of Adult and Pediatric Astrocytomas
Source: PLoS One. 2012 Aug 22;7(8):e43339. doi: 10.1371/journal.pone.0043339 (PMC3425586; doi:10.1371/journal.pone.0043339)
Supplement: Figure S5 — Expression of survival-associated immune response-related genes in glioma infiltrating microglia/macrophages relative to bulk tumor. (PDF) [file pone.0043339.s005.pdf]

Engler et al, 2012, PLoS ONE

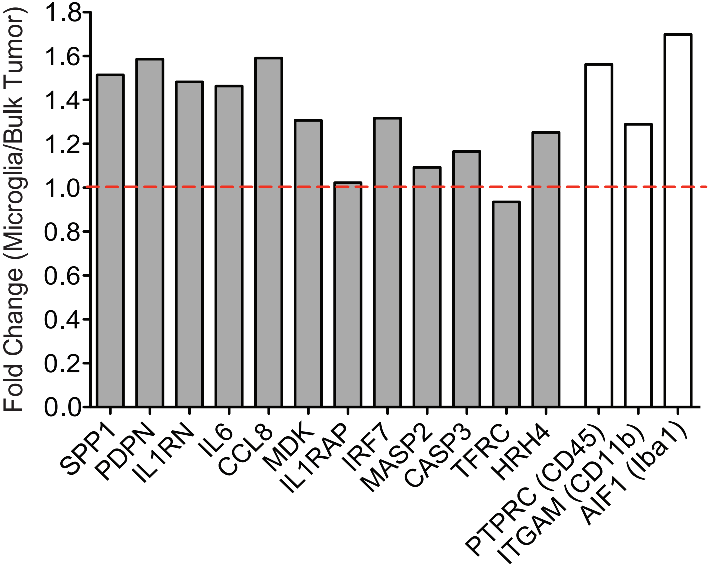

Figure S5. Expression of survival-associated immune response-related genes in glioma infiltrating microglia/macrophages (GIM) relative to bulk tumor. Grey bars indicate immune genes identified by CoxBoost analysis (Table 1). White bars indicate common microglia/macrophage markers. The red dotted line denotes a 1-fold change, hence no difference in gene expression between GIM and bulk tumor.
