# Supplementary material for: Increased Microglia/Macrophage Gene Expression in a Subset of Adult and Pediatric Astrocytomas
Source: PLoS One. 2012 Aug 22;7(8):e43339. doi: 10.1371/journal.pone.0043339 (PMC3425586; doi:10.1371/journal.pone.0043339)
Supplement: Figure S6 — Correlation between microglia/macrophage cell number and hypoxia or vascularity in adult GBM. (PDF) [file pone.0043339.s006.pdf]

Engler et al, 2012, PLoS ONE

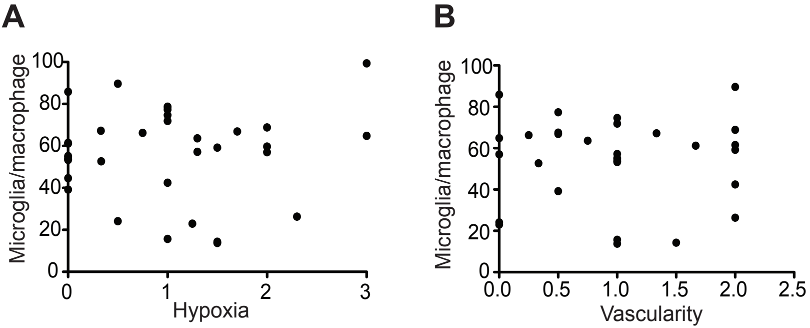


**Figure S6. Correlation between microglia/macrophage cell number and hypoxia or vascularity in adult GBM.** Lack of correlation between microglia/macrophage cell number (Iba1) and **(A)** hypoxia (CA9) and **(B)** vascularity (CD34) in adult GBM tumors.
